# Co-production of the ‘EPIC’ multidimensional tool-kit to support neurodivergent children and young people at home and school: a feasibility and pilot study

**DOI:** 10.1186/s40814-024-01530-3

**Published:** 2024-08-10

**Authors:** Sinead M. Rhodes, Emily McDougal, Christina Efthymiou, Tracy M. Stewart, Josie N. Booth

**Affiliations:** 1 Department of Child Life and Health, University of Edinburgh, Royal Hospital for Children and Young People, Little France, Edinburgh, EH16 4TJ Scotland; 2 Evidence Based Practice Unit, Anna Freud and UCL, London, UK; 3 Moray House School of Education and Sport, University of Edinburgh, Edinburgh, EH8 8AQ Scotland

**Keywords:** Psychoeducation, Executive functions, Co-productions, Strengths and difficulties, ADHD, Autism

## Abstract

**Background:**

Interventions focused on cognitive function in neurodivergent children typically focus on single functions, e.g. working memory training. They are often focused on ‘deficit’ models and lack an emphasis on understanding areas of individual strengths and difficulties as a prerequisite to appropriate support. The multidimensional nature and phenotypic variability of cognitive profiles in these children indicate a need for a multicomponent-tailored intervention programme focused on understanding and supporting an individual child’s cognitive functioning.

**Aims:**

The ‘EPIC’ intervention (Edinburgh Psychoeducation Intervention for Children and Young People) is focused on improving cognition, learning and behaviour in neurodivergent children such as those with attention deficit hyperactivity disorder (ADHD) or who are autistic. Building on our previous co-production work, this study aimed to use a participatory methods approach to develop EPIC practices and materials in relation to our key principles which include psychoeducation, multicomponent, individualised approach, strengths and difficulties profiling and pairing of a child’s individual strengths and difficulties with internal and external strategies. We also set out to assess the feasibility and acceptability of EPIC, and pilot this novel tool-kit intervention with neurodivergent children and their parents and teachers.

**Methods:**

The intervention practices, materials and strategies of EPIC were co-produced with neurodivergent children, their parents, teachers and clinicians taking a strengths and difficulties approach. Identification of psychoeducation activities and strategy practices (e.g. mind-maps, chunking), testing of feasibility and collection of pilot data were conducted over a bi-weekly 8-week programme. Eleven neurodivergent children aged 7 to 12 completed the 16-session individualised programme. Acceptability and feasibility were ascertained via qualitative reports elicited within child and teacher interviews and child ratings of enjoyment. Pilot evaluation data was collected pre- and post-intervention participation, and across cognitive assessments (CANTAB, BRIEF), educational attainment (WIAT) and parent and teacher questionnaires measuring clinical symptoms and behaviour (Conners, AQ, SDQ, self-perception). Data was compared with a matched neurodivergent treatment-as-usual control group (*N* = 9).

**Results:**

The co-produced EPIC intervention was both feasible to deliver and acceptable to children, parents and their teachers. Pilot data identified that the 8-week intervention improved cognition (short-term and working memory) and literacy (receptive vocabulary, oral word fluency, listening comprehension). Improvements in the intervention group were also found for parent-reported child behavioural difficulties and aggression, and teacher-reported scholastic competence. Effect sizes generated (Cohen’s d) ranged from 0.65 to 2.83. Parents reported continuing to use EPIC strategies when interviewed over a year after participating in the programme.

**Conclusion:**

The current study met our objectives fully. ‘EPIC’ (Edinburgh Psychoeducation Intervention for Children and Young People) is feasible in home and school contexts and improves a range of aspects of cognition, learning and behaviour in neurodivergent children. Our findings show EPIC is suitable to be assessed within a full-scale trial.

## Key messages regarding feasibility

- Co-production of an intervention is feasible with neurodivergent children, their parents and teachers.
- The EPIC intervention is feasible in home and school settings and is acceptable to children, parents and teachers.
- The EPIC intervention activities are enjoyable for children.

## Background

Neurodevelopmental diagnoses, including autism^1^, attention deficit hyperactivity disorder (ADHD) and developmental coordination disorder/dyspraxia (DCD) are very common, and prevalence rates have been increasing over time [2, 3]. The conditions commonly co-occur and these children (also known as neurodivergent children) often share cognitive, learning and behavioural profiles leading researchers to take a transdiagnostic approach when conducting research in this area [4, 5]. Neurodivergent children often present with difficulties in cognitive skills that impact their academic achievements [6–8]. For example, children with ADHD often have difficulties in inhibition, memory, planning and decision-making [9, 10], and children with DCD and autistic children also have similar difficulties in executive functions [11–14].

Research has shown that the thinking differences these children show are linked to educational attainment both in relation to mathematics learning [7] and literacy difficulties [8]. Neurodivergent children are also at high risk of mental health problems including those with DCD [15], ADHD [16] and autistic children [17]. The development of appropriate interventions to target cognitive difficulties is essential, with optimal delivery being from early in childhood from which point these difficulties are often first observed. Neurodivergent children show heterogeneous cognitive profiles though [18–20] highlighting the importance of the development of interventions that can be tailored to an individual child’s particular strengths and difficulties.

Researchers have identified a range of cognitive factors associated with academic learning in neurodivergent children [6–8]. Cognitive functions, used to organise our behaviour such as working memory, inhibitory control, planning, strategy use, and attention flexibility, known collectively as executive functions, have in particular been implicated [9, 10, 21–26]. Research has identified three core aspects of executive function [27]. Miyake et al. [27] identified: shifting, updating (more generally known as working memory) and inhibition that are moderately correlated but separable. Diamond [28] identified planning as an additional important aspect of executive functioning. Research has strongly implicated difficulties in working memory in neurodivergent children, a system wherein information is stored and controlled in memory [29, 30]. Broader aspects of executive function, such as self-regulation and meta-cognition, also cause difficulties for some children’s learning — for example by impeding preparatory processes and self-monitoring of task performance [22].

Many neurodivergent children, however, thrive in aspects of cognitive function and learning [31, 32]. Neurodivergent characteristics such as special interests, impulsivity and hyperfocus can have a positive impact on learning and attainment across the lifespan [33, 34], but many neurodivergent children often also show academic learning difficulties in other areas from early in life. Children with ADHD begin to show learning difficulties from early in development, as early as 3 years of age for many children [35]. Young people with ADHD are at greater risk for adverse educational outcomes including academic underachievement, repetition of school years and higher dropout rates [36]. Cognitive difficulties in ADHD and DCD have also been linked to poor academic learning outcomes [37, 38]. Furthermore, autism has been linked to reading comprehension difficulties [39] and DCD has been linked with difficulties with literacy and mathematics [21, 40]. Literacy difficulties in ADHD are broad and extend beyond reading [41] to include spelling [37] and written expression [42]. Many children with ADHD also show difficulties in mathematics learning [43]. A population-based study suggested that children with high DCD symptoms have similar academic difficulties to children with ADHD; adolescents with DCD achieved two GCSEs while their peers (matched on SES, sex and age) achieved seven [38].

These difficulties, if not addressed, are likely to have life-long consequences for the child [44]. Research suggests that early mathematics comprehension is the principal predictor of later academic and socioeconomic outcomes in typically developing samples when assessed in adulthood [45, 46]. Reading difficulty in ADHD has been associated with poor socio-emotional functioning [47]. The similarity of cognitive and academic learning difficulties in different groups of children diagnosed with neurodevelopmental disorders [4] and their high rates of co-occurrence lead us to take a transdiagnostic approach in developing and testing an intervention to improve these outcomes.

Despite the broad range of cognitive difficulties experienced by neurodivergent children, most cognitive interventions have focused exclusively on working memory [48]. There is some evidence to support training interventions with neurodivergent children focused on remediating ‘working memory’ [49]. Despite this, results from intervention programmes such as ‘Cogmed’ have had mixed success [48, 50]. Small effect sizes are common, as well as the limited success of effects transferring beyond the type of task used in the study or to long-term effects beyond the end of the programme [51].

Research characterising cognitive function in neurodivergent children strongly suggests that it is important for interventions to focus on strategic cognitive difficulties beyond working memory that impact academic learning, including poor planning and self-regulation [6, 22, 23, 26]. These broader executive function skills are central to successful task initiation and completion but surprisingly have received little attention in intervention programmes. An intervention, ‘Unstuck and On Target’, developed with a broader set of aspects of cognitive function for autistic children has shown some success [52] and another developed for adolescents with epilepsy has similarly reported positive impacts on cognition and quality of life [53, 54]. With high rates of co-occurrence and heterogeneity of function within neurodevelopmental disorders though, it is imperative that a multicomponent intervention is developed that enables an individualised approach for neurodivergent children. We designed the intervention ‘EPIC’(Edinburgh Psychoeducation Intervention for Children and Young People) to address these gaps.

### Key underlying principles and elements of EPIC

The development of EPIC principles was based on the MRC framework for developing and evaluating interventions [55]. These guidelines focus on six core elements: intervention context, underpinning theory, inclusion of diverse stakeholder perspectives, identifying key uncertainties, intervention refinement and comparative resource and outcome consequences. Alongside a multicomponent, individualised approach being vital to EPIC, we identified other elements from the literature including strengths and difficulties profiling, psychoeducation and pairing of a child’s individual strengths and difficulties with internal (e.g. rehearsal, chunking) and external (e.g. timers, mind-maps) strategies. Historically, intervention research with neurodivergent children has focused on models of ‘deficit’. While we know that neurodivergent children often show significant difficulties in areas such as cognition and learning, there is also evidence that many show strengths in these aspects of functioning [31, 32]. Most interventions to date have focused on areas of difficulty such as in working memory processing without reference to strengths. There is a need for intervention development to ascertain if strategies focusing on a child’s individual strengths can help overcome areas of difficulty.

The importance of psychoeducation, ensuring the child and adults who support them understand the individual child’s strengths and difficulties, has been emphasised repeatedly in the literature [56, 57]. The National Institute for Health and Care Excellence (NICE) clinical guidelines indeed recommend the inclusion of psychoeducation in the treatment plan for children with ADHD and their families [58]. A survey conducted by Children in Scotland, The National Autistic Society of Scotland, and Scottish Autism [59] of 1417 parents of children with autism revealed a range of school-based factors that parents prioritised as making a difference in their children’s lives. The most highly endorsed item was ‘school staff having a better understanding of how their child’s autism affects them’. While psychoeducation has become an increasingly important component of clinician post-diagnostic support, it has received little focus within education. Existing interventions have focused on strategies — there is a need to develop an intervention that includes psychoeducation as a core practice, to enable the neurodivergent child to understand the particular cognitive difficulties causing them challenges in school work and home life. Once understood they can then be paired with optimal strategies to support a child’s difficulties.

Over the last 50 years or so, the cognitive psychology literature has identified a broad range of techniques that can support cognitive functioning. Many of these strategies are focused on memory processes such as rehearsal [60], chunking [61] and use of interactive imagery [62]. The development of the current intervention was based on the premise that once difficulties are profiled and understood, they can be paired with the use of these internal strategies to support them. In developing the EPIC intervention in practice, we also aimed to identify if the use of external strategies that are similarly focused on supporting cognitive processes (e.g. mind-maps, timers, physical planners) could be used to improve cognitive function in neurodivergent children. We planned to take a game and activity-based approach with children being active participants in their learning. Active participation in learning has been documented to be an important factor in determining learning outcome success [63].

Following the identification of these key principles and elements, and using the MRC guidance as a framework [55], we aimed to identify how the intervention components could work in practice, alongside the investigation of the feasibility of EPIC. We also set out to examine the effectiveness of EPIC through collection of pilot data with a particular focus on interpretation of effect sizes. We initially planned a three-arm trial with an intervention group, a drug naïve treatment as a usual neurodivergent group and a medication group. COVID-19 impact on medication titration appointments led to having to drop the medication arm of the study. We, therefore, conducted a two-arm intervention programme comparing children who received a bi-weekly 16-session intervention over 8 weeks with the non-intervention treatment as usual group.

This study had the following objectives:

1. To develop intervention components in practice including materials, psychoeducation practices and strategies (development in practice);
2. To investigate if an 8-week researcher-led programme of EPIC is feasible (assessed via child and teacher qualitative data) and acceptable (assessed quantitatively) in school and home contexts, and if recruitment, uptake, and participation completion is feasible (feasibility and acceptability);
3. To collect pilot data on the effectiveness of EPIC and generate effect sizes to inform a definitive trial. We examined the impact on cognitive function, academic learning, and behaviour alongside knowledge about neurodiversity in a small sample of children (pilot study).

## Method

### Development in practice

To ensure we achieved the inclusion of ‘diverse stakeholder perspectives’ [55], we refined the core elements of EPIC through Patient and Public Involvement (PPI) work using a co-production approach in a previously published study [64]. We interviewed neurodivergent children and their teachers in a co-production study to inform how the EPIC principles could be developed in practice in the form of an intervention [64]. This study revealed that children had very limited knowledge of their condition and teachers’ knowledge often centred on core symptoms and behaviour. Knowledge about broader areas of difficulties such as executive functions was limited. This fits with areas of research need identified by research priority exercises. A James Lind Alliance ‘learning difficulties’ exercise highlighted that the top stakeholder priority for research with these children is identifying the knowledge and training teachers need to provide optimal support and achieve the best outcomes [65]. We therefore planned to develop EPIC as a school-based intervention with child and teacher sessions.

The interviews we conducted with neurodivergent children and their teachers [64] were complemented by workshops with education professionals, parents and clinicians [66, 67]. We consulted our stakeholder group of educational professionals and parents that was set up to influence our research priorities, practices and outputs more generally. In a knowledge exchange workshop, we shared findings from our research which teachers could use for their continued professional development (their preference for how to be acknowledged), and in the second half of the workshop, stakeholders shared their views on the booklets. A neurotypical parent of a neurodivergent child, a neurodivergent teacher who taught neurodivergent children in school and a neurotypical teacher took part in an online interview. The stakeholders reviewed each section of the booklets to ensure the language was sensitive, the layout was clear, and the strategies provided were helpful in the context of the classroom environment.

This work enabled us to extend items to the list of strategies collated by the research team and ensure materials were accessible, activities were feasible, and language was appropriate. We co-produced ‘understanding’ neurodevelopmental condition booklets and a linked ‘strategies’ booklet with relevant teachers and other education professionals alongside clinicians and parents. These booklets^2^ became central to the intervention activities. The EPIC intervention advocates focusing on children’s strengths to help resolve thinking difficulties. Our PPI work identified ‘personalisation’ to be an important factor here. Drawing on children’s special interests and preferred toys and objects was identified as an area of strength for children in overcoming areas of difficulty. In our interviews with teachers [64], they described creativity and imagination as being areas of strength for neurodivergent children and importantly commented that these skills were sometimes applied in academic learning such as creative writing in literacy.

**Fig. 1:**
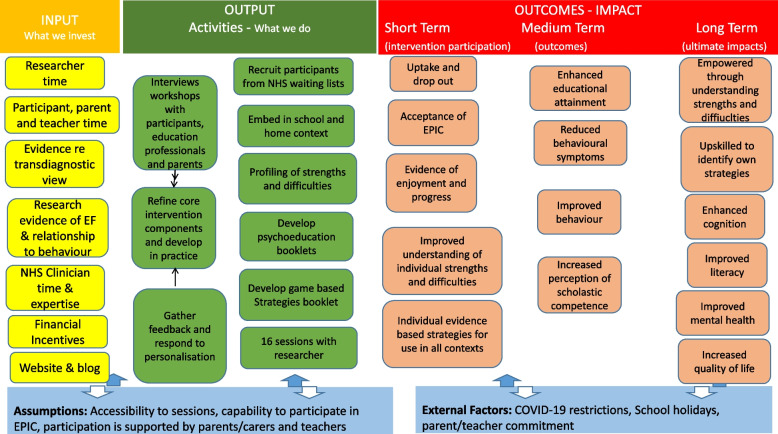
Details the logic model for EPIC

Strategies included in EPIC focus on attention, inhibition, planning, memory, self-regulation, emotional regulation and wellbeing — these are detailed in the ‘strategy’ booklet. The ‘understanding’ booklets are linked with the strategies, pairing an understanding of the child’s individual thinking difficulties with appropriate strategies such as ‘chunking’, ‘mental imagery’, ‘mind maps’, use of diaries or planners and ‘stop and think’ strategies. During this development in practice phase, intervention elements undertook further refinement identifying ways in which the underlying intervention principles could work in practice Fig. 1.

### Feasibility, acceptability and pilot testing

Following the initial development of EPIC, we undertook feasibility and pilot testing to address objectives 2 and 3. We also undertook further intervention refinement across this phase responding to the views of children, parents and teachers and researcher perspectives.

### Participants

We recruited children who were on NHS Child and Adolescent Mental Health Services (CAMHS) Neurodevelopmental Disorder pathway (for ADHD assessment) waiting lists in NHS Lothian, UK. We aimed to recruit 20 children in each intervention group. As this was a pilot study, a sample size calculation was not performed [68]. We aimed for 20 participants in each group because we felt this would sufficiently inform us of the practicalities of delivering the intervention and produce indicative effect sizes alongside qualitative feasibility data and acceptability [68]. 

CAMHS clinicians distributed information packs to children on the waiting list who met inclusion criteria and did not meet exclusion criteria with every other child assigned either to the intervention (final *N* = 11) or control, i.e. treatment as usual (final *N* = 9) group. The inclusion criteria were as follows: male or female aged between 6 years 0 months and 12 years 11 months on the waiting list for Neurodevelopmental Disorder Assessment. The exclusion criteria were as follows: the presence of a known intellectual disability, children with a known chromosomal condition or uncorrected vision or hearing impairment, children who had previously or currently taken stimulant medication. All children had high ADHD symptoms (Conners T score > 70) and many had high co-occurring autism and/or DCD/dyspraxia symptoms or diagnoses in some cases. Following the transdiagnostic approach EPIC takes, we refer to the sample as ‘neurodivergent children’ (see Table 1 for participant characteristics). The recruitment method of an ADHD pathway was chosen to ensure children were drug naïve and not exposed to medication as this is known to improve cognitive function [25]. Favourable ethical opinion was granted from the North East York NHS Research Ethics Committee (REC reference: 19/NE/0267). All children were given an opportunity to provide verbal (ages 6–8 years) or written (ages 9–12 years) assent at the first visit. Child assent and parent and teacher consent were managed by the provision of separate information sheets and secured during a face-to-face meeting to ensure fully informed assent. Parents of children provided full informed written consent for their child’s participation, as well as their own participation.

**Table 1:** Participant profiles (means unless otherwise stated, S.D.)

	Intervention group (*n* = 11)	Control group (*n* = 9)
Age at baseline (months)	99.55 (15.85)	94.33 (17.48)
Sex	8 M, 3 F	6 M, 3F
Socio-economic status (SIMD)^a^	3 (1.48) Range: 1–5	2.89 (1.54) Range: 1–5
Birth weight	3281.22 g (543.41)	3396.55 g (880.06)
Gestational age	39.44 (1.88)	38.78 weeks (1.72)
Handedness	1 left-handed	All right-handed
WASI full-scale IQ composite score	91.50 (12.23)	95.44 (13.18)
BPVS percentile rank	43.14 (25)	37.11 (18.72)
ADHD symptoms: (Conners Parent Inattentive T score)	83.1 (8.19)	83.33 (8.59)
ADHD symptoms: (Conners Parent Hyperactivity T Score)	84.2 (8.35)	81.56 (8.81)
AQ (% at or over criteria suggested for referral for autism assessment)	27.27%	33.33%
DCD (M-ABC2) (% in ‘red’ zone)	45.45%	22.22%
SDQ (total score)	19.7 (4.11)	23.22 (9.0)

*SD* Standard deviation, *n* Number of patients with data available

^a^Socioeconomic status (SES) is also known to impact educational outcomes; therefore, the SES of each child was calculated using the Scottish Index of Multiple Deprivation (SIMD), which is an area-based measure of relative deprivation. The child’s home postcode was entered into the tool which provided a score of deprivation on a scale of 1 to 5. A score of 1 is given to the 20% most deprived data zones in Scotland, and a score of 5 indicates the area was within the 20% least deprived areas

### COVID-19-related changes to procedures

The original design of the study was focused on school administration, and therefore, parents did not initially take part. However, in response to the COVID-19 lockdown of 2020, and following approved amendments from the ethics committee, parent intervention sessions were incorporated into EPIC. Parent sessions took place in March 2021, which was the first opportunity to visit homes following the COVID-19 lockdown. This enabled us to develop an intervention that can be embedded across both school and home settings. Parent insights were sought informally following the intervention completion.

In addition to the young people, both parents and teachers participated in sessions meaning the intervention programme outcomes were embedded across contexts.

### Measures

#### IQ

Children completed the Wechsler Abbreviated Scale of Intelligence (WASI-II) [69] and the British Picture Vocabulary Scale (BPVS-III) [70]. The WASI-II has been used extensively as a test of intelligence in clinical child populations and shows high correlations with the more comprehensive equivalent of the Wechsler Intelligence Scale for Children [4]. Four subtests were administered in sequential order: Block Design, Vocabulary, Matrix Reasoning and Similarities. A raw score was calculated for each subtest and converted to a T score using age-standardised norms. This measure has good test–retest reliability for child samples (*r* = 0.96), and the FSIQ-4 scores from this measure correlate with the Wechsler Intelligence Scale for Children–Fifth Edition (WISC-V) [71], *r* = 0.87 [72] indicating good concurrent validity. The BPVS-III was used to provide an index of receptive vocabulary IQ. It is a standardised measure used to assess verbal ability in children with neurodevelopmental disorders [73]. Reliability has been reported to be 0.91 [70]. Raw scores were converted into age-standardised scores.

#### Neurodevelopmental symptom questionnaires

Questionnaires were completed by parents/carers following standard administration according to test manuals. Child behaviour data was collected using the Strengths and Difficulties Questionnaire (SDQ) [74] which consists of 25 items measuring five broad constructs: emotional problems, conduct problems, hyperactivity, peer problems and prosocial behaviour. The SDQ has good test–retest reliability (*r* = 0.70) and internal consistency (*α* = 0.73) [75–77]. The Conners 3-Parent [78] 110-item questionnaire was used to provide a dimensional assessment of ADHD and related difficulties in children and adolescents. The Conners has previously been used to assess ADHD symptoms in children [5, 79]. The Conners has good internal consistency (*α* = 0.97) and test re-test reliability (*r* = 0.98) and is used routinely in clinical settings [78]. Parents completed the Autism Quotient (AQ) which was used to measure child autism symptoms [80]. We initially used the AQ10 (*N* = 9) and then changed it to AQ50 (*N* = 11). The AQ has good internal consistency (*α* = 0.97) and test–retest reliability (*r* = 0.85) [80]. The Movement ABC-2 Checklist [81] was used to measure DCD/Dyspraxia related symptoms. The Movement ABC-2 Checklist measures functional motor performance in children aged 5–12 years and has been reported to have good internal consistency (*α* = 0.94) [82]. Higher scores on these questionnaires indicate poorer function/higher levels of symptoms.

#### Self-perception questionnaires

We also included child self-report and teacher-report measures to identify perceptions of change which may not have been fully observable statistically on objective outcome measures after 8 weeks. The Self-Perception Profile [83] was completed by children (36 items) and teachers (15 items). This scale measures scholastic competence, social competence, global self-worth and behavioural conduct in school. The Self-Perception Profile has good internal reliability (*α* = 0.83–0.95) [84].

#### Cognitive tasks/questionnaires

Children completed five tasks from the new version of the Cambridge Neuropsychological Test Automated Battery (CANTAB) [85]. Tasks included were the Spatial Span Forwards and Backwards Memory task, the Spatial Working Memory task, the ID/ED attention shifting task, the Stockings of Cambridge (SOC) planning task, the Stop Signal inhibition task (SST) mapping on to the four key components of executive function namely working memory, cognitive flexibility, planning and inhibition. Spatial Span Forwards assesses non-EF short-term memory span and the backwards version assesses working memory with an EF component. The SWM task was included as it includes an updating component putting a heavy load on EF. The Delayed Matching to Sample (DMtS) short-term memory task was employed as a non-executive function memory index. This task involves holding information in memory over a delay and neurodivergent children have been shown to have significant difficulties with this task [10]. The game-like format of these tasks and completion on a touch screen iPad make them highly suitable for children with Neurodevelopmental difficulties. Together, these tasks tap areas of memory and executive function known to often be impaired in neurodivergent children.

In addition, teachers of the intervention group children completed the Teacher BRIEF-2 executive function questionnaire [86] for pre- and post-test evaluation and for use in profiling. The BRIEF-2 has good internal consistency with coefficients ranging from 0.88 to 0.98, with index and composite scores ranging from 0.94 to 0.98 and good test–retest reliability with correlation coefficients ranging from 0.76 to 0.89 (*M* = 0.82) [87]. The use of the BRIEF-2 enabled an index of executive functions that added ecological validity to the objective cognitive data.

#### Educational tasks (WIAT)

Children were assessed on educational tests from the Weschler Individual Achievement Test (WIAT-III) [88] including tests of word reading, reading comprehension, spelling, written expression, listening comprehension and oral expression, mathematical operations and reasoning. The WIAT has good to excellent (0.83–0.97) reliability for subtests and good validity (0.62–0.80) [89].

#### Perceived enjoyment and progress

Children provided ratings of enjoyment and their perception of their progress of each strategy application on a printed table constructed by the research team. A 1–5 Likert rating scale ranging from low to high was used to rate enjoyment and perceived progress.

### Procedure

Children were recruited from three CAMHS teams in Lothian. CAMHS teams sent out information packs to children on the waiting list who met inclusion criteria and interested participants returned a contact consent form back to the research team. Pre- and post-intervention testing was conducted by a research psychologist and typically carried out across an initial home visit and then two school visits usually a week apart. Sessions were planned to be 45 min in length but ranged from 30 min to 1 h duration depending on breaks and home and school constraints. All children undertook cognitive and educational testing at time 1 and then the same testing battery 8 weeks later at time 2. Parents, teachers and children completed a range of questionnaires to measure behaviour, wellbeing, cognition and ADHD symptoms. Participants in the intervention arm took part in a 16-session programme of EPIC over 8 weeks, whereas the usual treatment control received no intervention.

#### Intervention sessions

Intervention participants, their parents, and teachers completed the measures described above within a 2–3-week period prior to starting and completing the 8-week intervention. Intervention sessions were largely conducted by CAMHS psychiatrists undergoing higher research training under the supervision of the first author or alternatively by a research psychologist qualified to the post-graduate level. Sessions most frequently took place at home (mean 2 sessions) or school (mean 6.1) but sometimes were online (mean 3) or via a phone call (mean 2.3). Phone and online calls were introduced as an option during COVID-19 restrictions. Home visits were introduced as an option during the COVID-19 pandemic in 2020 but were not possible for participants 6 and 7 due to COVID-19 home visit restrictions in 2021.

All tasks (pre-intervention) were used for profiling to inform the cognitive processes that would be focused on within the 16 sessions. Some additional measures (indicated below) were also used for profiling. Control participants completed baseline measures without those required solely for profiling.

A number of documents were constructed to help with profiling the child’s strengths and difficulties: a ‘learning log’, an ‘about me’ and a ‘strategy record’. These were used to record information about the child’s thinking skills and learning.

Teachers and children took part in an interview pre- and post-intervention to ascertain ADHD knowledge and strategy use. The interviews also set out to document improvements as well as barriers and facilitators to intervention delivery. Five of the intervention children participated in pre-intervention interviews and five in post-intervention interviews. Ten teachers took part in at least one interview.

Intervention sessions were typically 45 min duration but ranged from 15 min to 1 h according to child, parent and teacher factors and restrictions. Sessions were mainly conducted face-to-face but were conducted online during COVID-19 restriction periods. Sessions 1 and 2 focused on psychoeducation although dialogue around understanding the child’s neurodevelopmental difficulties was continued across all sessions. Depending on the child’s profile, a range of strategies were chosen to focus on and activities outlined in the booklets were conducted with the child to deliver continued psychoeducation and to pair with suitable strategies. Teacher and parent sessions focused on the same areas aiming to upskill the parent and teacher to understand the child’s individual strengths and difficulties and to engage in activities that support them. The aim of EPIC is to support the child to practice strategies and then use these strategies in the classroom (and other contexts). The programme aims then to continue to have an impact beyond the sessions delivered by the researcher. Reporting has been influenced by the TIDieR checklist [90].

## Results

### Feasibility

#### Recruitment

**Fig. 2:**
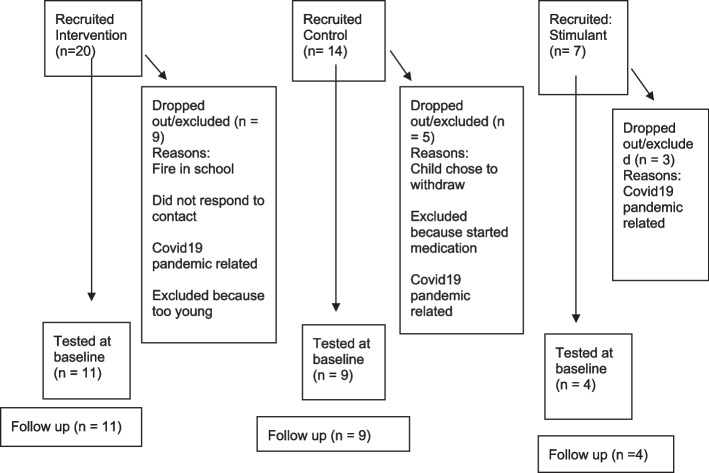
Flow chart showing participant recruitment, dropout and follow up

We successfully recruited 24 children to take part in EPIC and undertake pre and post testing (see Fig. 2 for a flowchart detailing numbers from recruitment to end completion numbers).

Recruitment take-up data across groups revealed that 1 in 3 families who were approached signed up to take part. Forty-one families were recruited, two of which were identified as ineligible following initial screening. We recruited 20 to the intervention group, 14 to the control and 7 to the medication group. Drop-out was due to COVID-19 restrictions, with the exception of two children who could not take part due to a significant fire in the school. This meant a final sample of 11 in the intervention group, 9 in the control group and 4 in the medication group. Over 50% of participants initially recruited completed baseline testing and 100% of participants who completed baseline testing completed follow-up testing which is in line with criteria for progression reported in other studies [91]. Challenges linked to recruiting the medication group were COVID-related arising from increased gaps between diagnosis and medication titration. This extended waiting list times which we had not anticipated in our pre-pandemic design led to a completed sample of *N* = 4. Data analysis was therefore not conducted with this group.

#### Participation

Children in the intervention arm typically received 8 sessions (range = 5–8, *M* = 7.1) focused on psychoeducation and strategies, and we also sought to conduct 8 sessions with their parent and/or teacher (teacher range = 0–5, *M* = 3.4; parent range = 0–7, *M* = 3.3). All children who took part in baseline testing in the intervention and control groups took part in follow-up testing. The rate of completion of tasks (completing all cognitive and educational tasks within testing) within baseline and follow-up child testing was generally high across the three groups. In the intervention group, 9 out of 11 (82%) children completed all baseline and follow-up post testing, while 7 out of 10 (70%) children completed all baseline and follow-up testing in the control group and in the medication group, all four children completed all baseline and follow-up testing.

We originally aimed to begin with researcher-led sessions with the child and then incorporate teachers conducting these sessions with the child. Two teachers attended child sessions; however, COVID restrictions from March 2020 meant that additional teachers were not able to. The aim of the sessions with teachers was to undertake psychoeducation about the individual child and provide the teacher with strategies to use with the child which were separate from sessions with the child. Teacher engagement was generally high, with teachers participating in an average of 3.3 sessions (ranging from 0 to 5 across teachers). Missed sessions arose from time capacity restrictions. Although the study initially set out to run all sessions in schools with teachers, COVID-19 restrictions meant we extended sessions to parents and this was a valuable aspect of the study. Parental engagement was also high with parents taking part in an average of 3.4 sessions ranging from 0 to 7 sessions across parents.

#### Perceived enjoyment and progress

Child ratings of enjoyment and their perception of their progress of each strategy application increased from the first 2 to the last 2 sessions. A paired sample *t*-test revealed that children’s enjoyment ratings in sessions 1 and 2 (mean = 3.6) and at later sessions (mean = 4.6) showed an increase in enjoyment over time (t(1,5) =  − 2.14, *p* < 0.05). In terms of perceived progress, participants reported an average score of 3.9 out of 5 for strategy proficiency at earlier sessions and 4.4 out of 5 at later sessions (sessions 7 and 8), suggesting an increase in perceived ability over time although this was not statistically significant (t(1,5) =  − 1.168, *p* > 0.05).

#### Evaluation based on participant insights

##### Teachers

During pre-intervention interviews, most teachers showed an understanding of ADHD based on behaviour (e.g. fidgeting, hyperactivity). Strategies they focused on pre-intervention were most often focused on academic skills (e.g. phonics). Post-intervention several teachers showed a much more comprehensive understanding of ADHD with more reference to cognition at post interview. One teacher commented, ‘I always thought it was a behavioural thing but I supposed I realised it is a biological thing. I hadn’t really thought about it from an organisational point of view’. This teacher also commented on improvement in the strategies the child was using post-intervention, ‘I am thinking of strategies some sort of regulation thing, is working better for him. I noticed that he has been able to come up with a few strategies himself with self-regulation’.

Another commented, ‘The pupil has made a lot of progress since she started the research study. She has been using many checklists in the classroom and she is more organised. The project has been very useful to her and the pupil has started doing a lot of things she used to find difficult in the past such as lists and using the white board in school’.

##### Children

The two children who did both pre- and post-intervention interviews mentioned the use of strategies in the post interview session. In this second interview, they mentioned for the first time reference to mind maps, timers, taking breaks and fidget suggesting they had become more aware of these strategies and how to apply them.

##### Parent perspectives

While we did not formally collect qualitative data with parents in this work, we did receive feedback from parents during and after their involvement in the intervention e.g. ‘The work done by the researchers was really relevant for my child’s understanding about ADHD. My child is also making use of the strategies presented by the researcher and these strategies are helping my child to have some more control of her emotions and impulsiveness. Although my child still has a lot to learn about ADHD, she is more aware now that she needs to use some strategies to be able to achieve some goals more quickly and efficiently’.

Parents also made specific comments during the intervention sessions that suggested an improved understanding of ADHD and the use of strategies. For example, when asked if he thought he could have ADHD at the first session, one child responded ‘definitely not’. By session 6, his mother said he had a greater understanding of ADHD and his own particular difficulties saying, ‘he now often says my brain is really busy at the moment’ and inferred that this better articulation of his ideas helped him to cope better at school.

### Barriers and facilitators

Using a tool-kit approach, we identified areas of cognition and other aspects according to the individual child’s profile. This identified that some children needed to focus on a greater number of aspects of function than others in the intervention. The number of different aspects of cognition (e.g. memory) or behaviour/other (e.g. emotional regulation) the researcher focused on had a mean of 4.9 and ranged between 3 and 6 areas focused on. Diarised contextual facilitators to intervention delivery identified by researchers (R) and teachers (T) included reminding the child what happened in the last session (R), short sessions of no longer than 45 min (T) or two 30-min rather than a 1-h session with a teacher per week (T), conducting some sessions in the classroom (T and R), use of EPIC resources facilitated engagement and practice of strategies (T), use of strategies in a whole class approach (T), inclusion of parent in-home sessions (T and R), use of active leaning activities (T and R), use of materials easily available, e.g. lists, whiteboards, timers and fidget toys (T and R). Diarised contextual barriers to intervention delivery included the use of 1–2-1 games within the class setting (T), sessions over 45 min (T and R) and change of researcher doing sessions (T) (caused by new employment).

COVID-19 lockdowns meant that we moved to online sessions at stages. This enabled us to show that intervention sessions could be conducted with teachers online and many reported that the additional flexibility of providing this option facilitated their ability to engage with the intervention. We also tested the feasibility of conducting online sessions with children and found that this worked well and was feasible.

#### Pilot data

The pre-post change data from the intervention (*N* = 11) and control group (*N* = 10) were compared using independent *t*-tests. Mean values, t-statistics and Cohen’s d effect sizes for significant differences and trends are shown in Table 2. A *p* value of 0.05 was taken as evidence of statistical significance with *p* values between 0.05 and 0.07 taken as evidence of a trend. Effect sizes (Cohen’s d) are reported following the MRC framework to inform a large-scale trial and interpreted as 0.2 = small; 0.5 = medium and 0.8 and greater as large.

**Table 2:** Data from a pilot study (means, S.D.)

Measure	Baseline intervention	Follow-up intervention	Baseline control	Follow-up control	Effect size	Confidence intervals
SWM between Search errors	23.45 (7.26)	16.64 (7.71)	22 (5.87)	21.29 (9.01)	*d* = 1.07	− .14, 13.49
DMtS % correct Delay	58 (31.9)	70.91 (27.33)	60 (31.62)	57.14 (24.3)	*d* = 1.01	− 55.77, .92
WIAT listening comprehension	94.36 (16.19)	102.36 (17.05)	101.67 (14.75)	91.14 (34.07)	*d* = .88	42.49, .78
WIAT receptive vocabulary	97.81 (16.19)	106.82 (15.85)	105.89 (12.50)	102.86 (8.88)	*d* = 1.06	− 26.51, − .35
WIAT oral word fluency	88.82 (29.17)	108.3 (20.63)	99.67 (9.53)	86.71 (17.09)	*d* = 1.37	− 45.8, − 5.25
WIAT expressive vocabulary	90.64 (10.58)	95.3 (15.77)	91.89 (12.05)	94.0 (14.09)	*d* = 1.36	− 73.55, 4.01
WIAT oral expression	86.45 (21.2)	95.44 (18.13)	93.67 (11.18)	89.86 (16.85)	*d* = .65	− 20.72, − 4.07
Conners parent learning problems	73.9 (15.41)	69.83 (13.99)	71.67 (13.38)	77.5 (10.49)	*d* = 1.94	− 4.79, 23.46
Conners parent aggression	76.4 (16.1)	66.83 (12.61)	73.67 (20.41)	82.13 (15.36)	*d* = .87	2.1, 33.11
Conners parent conduct disorder	65.80 (16.7)	59.83 (13.48)	66.89 (19.09)	73.13 (18.24)	*d* = 1.19	52, 25.43
Self-perception teacher scholastic competence^a^	2.0 (.0)	2.52 (.32)	N/A	N/A	*d* = 2.83	− .97, − .23

^a^Based on baseline/follow-up comparison in the intervention group only

### Descriptive data

The groups were not significantly different on age, sex, SIMD, birth weight, gestational age or handedness (all *p* > 0.05). They also did not differ on IQ (WASI Full Scale Composite Score) or vocabulary ability (BPVS Percentile Rank) (all *p* > 0.05). The groups also did not differ on mean symptoms of ADHD (Conners Parent), autism (Autism Quotient) or DCD (Movement ABC) (all *p* > 0.05). They also did not differ on any index of the Parent completed Strengths and Difficulties Questionnaire including emotional symptoms, peer relationships, prosocial behaviour and conduct problems (all *p* > 0.05) (see Table 1 for means and S.D.).

### Core outcome data

Independent *t*-tests were conducted on difference scores (follow up minus baseline data) comparing the intervention and control groups. Levene’s test for equality of variances was checked, and the t score was reported accordingly.

#### Cognitive data

There were significant differences and trends with the intervention group showing greater improvement in memory tasks. The intervention group showed a reduction in total errors on the Spatial Working Memory task (*p* = 0.05; CI − 0.14, 13.49) with a large effect size evident (Cohen’s d = 1.07). They showed a trend of greater accuracy on the Delayed Matching to Sample short-term memory task (*p* = 0.057; CI, − 55.77, 0.92) with a large effect size evident (Cohen’s d = 1.01) (see Table 2).

Paired *t*-tests revealed no significant changes on any of the indices of the teacher-completed BRIEF-2 questionnaire from pre- to post-test.

#### Educational data

There were significant differences between the groups on a number of literacy measures from the WIAT with the intervention group showing greater improvement than the control group on receptive vocabulary standard score (*p* = 0.045; CI, − 26.51, − 0.35) with a large effect size evident (Cohen’s d = 1.06). The intervention group also showed greater improvement in oral word fluency standard score (*p* = 0.017; CI, − 45.80, − 5.25) with a large effect size (Cohen’s d = 1.37) and oral expression (*p* = 0.006; CI, − 20.72, − 4.07) with a medium effect size (Cohen’s d = 0.65). There were trends for greater improvement from pre- to post-test for the intervention group compared to the control in listening comprehension (*p* = 0.058; CI, − 42.49, 0.78) with a large effect size evident (Cohen’s d = 0.88). There was also a trend for greater improvement in expressive vocabulary standard score (*p* = 0.075; CI, − 73.55, 4.01) with a large effect size evident (Cohen’s d = 1.36). There were no significant differences or trend differences between the groups on any of the maths assessments from the WIAT.

Teachers rated the intervention children higher in scholastic competence from pre- to post-test on the Self-Perception Questionnaire (t(1,4) =  − 4.47; *p* = 0.01; CI, − 0.97, − 0.23) with a large effect size evident (Cohen’s d = 2.83). There were no significant changes on any of the indices of the child completed Self- Perception Questionnaire from pre- to post-test.

#### Behaviour

Parent-rated child learning problems (*p* = 0.007; CI, − 4.79, 23.46) and aggression (*p* = 0.05; CI, − 2.1, 33.11) and conduct disorder (*p* = 0.043; CI, 0.52, 25.43) were reduced for the intervention compared to the control group over the 8-week period on the Conners questionnaire with large effect sizes evident (Table 2). There were no significant changes on any of the indices of the teacher completed Conners questionnaire from pre- to post-test. There were no significant differences or trends between the groups on the parent-rated SDQ.

### Follow-up interviews with parents (post participation in the EPIC programme)

We sought to undertake longitudinal follow-up interviews with teachers and parents post participation in the EPIC programme and secured 3 interviews with parents (14, 20 and 24 months post participation). These interviews led to useful insights of long-term impact and are summarised here. All parents expressed that the programme helped their children. The child’s self-concept was referred to as having benefitted from participation. Understanding that some of the difficulties they faced at school stem from their brain being different improved their perception of themselves (e.g. ‘She learned that if she has help she does better, she’s not stupid’). Participation also helped the children to stop feeling that it was their fault when they did not perform in school as well as their peers. The parents also noticed improvements in their child’s ability to undertake tasks (e.g. ‘he’s now able to follow my instructions’). The parents reported that they learned about neurodiversity during the programme. They perceived participation to be beneficial for family relationships. Understanding how the brain of their child was different changed some of the parents’ behaviours; this was illustrated by a mother who reported smoother interactions between her and her child as she expressed that she then understood her child better. Parents’ expressed improvements following psychoeducation; one parent reported that she used to worry about talking to her child about ADHD because she did not want the child to use it as an excuse to stop trying but now saw it as an explanation for her child’s struggles. She noted that it is now easier to discuss the condition with her child because they can focus on EPIC strategies. Parents reported they continued to use some of the strategies learned at home, e.g. chunking, stop and think and visual calendars.

## Discussion

This feasibility and pilot study fully met the objectives set. The three main practice elements, profiling, psychoeducation and pairing with strategies were further refined in relation to psychoeducation and strategy activities and materials used to support these. The co-produced EPIC intervention was both feasible to deliver and acceptable to children, parents and their teachers. Children showed an increase in their perceived enjoyment of activities across the 8-week period. Recruitment uptake was satisfactory for the intervention group and completion of intervention and control group participation was high, even given the challenging context of COVID-19 lockdowns. Pilot data identified variable effects across outcomes following the 8-week intervention; however, effect sizes ranged from 0.65 to 1.95 for outcomes where we identified statistically significant improvements, namely in cognition (short-term and working memory) and literacy (receptive vocabulary, oral word fluency, listening comprehension), albeit in a small sample. The parent reported child’s behavioural difficulties and aggression improved in the intervention group. Children and teacher’s knowledge of cognitive difficulties was broader post-intervention showing that psychoeducation had improved understanding. Follow-up interviews with a small sample of parents over a year later revealed perceptions of continued improvements in understanding and supporting children’s difficulties with strategies. The qualitative and quantitative pilot data show that EPIC improves a range of aspects of cognition, learning and behaviour in children with ADHD and should progress to be evaluated in a full-scale trial.

This study builds on other intervention studies that have taken a multicomponent approach. Kenworthy et al. [52] and Modi et al. [54] have reported on the effectiveness of interventions that focus on multiple aspects of executive functions with autistic children and children with epilepsy respectively. EPIC similarly takes a multicomponent approach going beyond executive functions to include broader aspects of cognition such as short and long-term memory and aspects of wellbeing such as emotional regulation. EPIC is also novel in taking an individualised transdiagnostic strengths and difficulties approach whereby the child’s individual profile informs the application of psychoeducation and strategies used. This ensures that a child’s actual rather than perceived difficulties are targeted. The approach also means that EPIC can be used for children without a diagnosis or who have these cognitive difficulties but that arise from a different cause such as epilepsy or prematurity. In the EPIC approach, the child is at the centre of the intervention and the focus is upskilling them to understand their own individual strengths and difficulties meaning they are empowered with the knowledge of their individual needs to help them in everyday situations. Cognitive interventions have frequently been criticised for showing limited success in any gains made transferring to other tasks particularly those involving far transfer such as in academic attainment [51, 92]. Over the 8-week EPIC intervention sessions, the researcher worked with the child to facilitate their understanding of strategies they could use and to identify the most appropriate strategy for their particular needs. In this approach, children are regarded as active agents of their own development where with a better understanding of their own strengths and difficulties they will be empowered to identify their own strategies to support themselves. Future research is required to identify if far transfer effects achieved by EPIC are sustained long-term beyond the parent interview data we collected and their perceptions of continued gain from intervention participation.

A central feature of the EPIC approach was working with children, parents, teachers and clinicians in the development of the psychoeducation approach, strategies and materials. The ideas generated via the PPI work added to the principles based on existing research to further develop the intervention. This co-production approach has undoubtedly facilitated the acceptability and feasibility of the EPIC intervention. Clinicians were involved in delivery of EPIC sessions leading to them influencing and contributing to the development of ideas around conducting psychoeducation and pairing with strategies within EPIC sessions.

There are a number of limitations in the current study. The COVID-19 pandemic posed various challenges including impacting drop-out and the form sessions could take for some participants. We had hoped to conduct sessions within classrooms but restrictions prevented this. The pandemic impacted the sample size we had hoped to recruit (*N* = 20 for each group). The pandemic also meant that some sessions were conducted via online video meetings. These sessions were conducted with both the parent and the child present, as opposed to in-person sessions that included the child on their own. Online sessions comprised the same material but the parent was involved in order to assist the researcher and engage with the material alongside their child. Online sessions with the parent and child enabled the delivery of the intervention material as the parent was able to observe for themselves the effect of the intervention on the child’s skills, as well as aiding the parent to learn and remember the session and possibly replicate this without the researcher in the future. Similarly, depending on the child, some participants were more willing to engage in the session when this was conducted with the parent. Online sessions with the parent and/or the teacher themselves had little difference with in-person sessions as the parents/teachers were presented with the same psychoeducation material both online and on paper. The adults were able to engage and interact similarly with the researcher on both occasions. In both the online and in-person versions, the intervention took an individualised approach to each child (e.g. use of personalised preferred materials) and so differences between parents are more generally a feature of the intervention.

The qualitative data provides important insights, but it is important to acknowledge the limitations here. Five of the intervention children participated in pre-intervention interviews and five in post-intervention interviews (only two of these children took part in both pre and post and therefore qualitative inferences of change are limited). Ten teachers took part in at least one interview (either pre- or post-intervention with four of these teachers taking part in both pre and post interview sessions). Nonetheless, the current study with a smaller sample size than planned reports significant improvements in the intervention group across areas of cognition, learning and behaviour. The sample size influenced us to take a descriptive and narrative approach to reporting feasibility [93]. Fundamentally, though, this was also determined by the co-production approach, we took to the development of EPIC where participants’ and stakeholders’ views are central to decision-making.

We developed all principles and elements of the trial prior to the study data collection commencement. As this is a trial conducted in a complex environment, we took a co-production approach to the specific activities/games/materials used that tested those elements and thus refer to this as ‘development in practice’. The use of a co-production approach invariably has an impact on reproducibility. This approach is needed given the individualised needs of these children and young people and the importance of children, parents and teachers selecting from a range of activities that best suits their needs and interests [94].

The findings are powerful in light of the fact that they reflect child performance on objective tasks as well as teacher interview data and parent and teacher questionnaire data. The EPIC programme was individualised to the profile of each individual child. While this likely maximised benefits gained by each child who participated, it makes it difficult to measure and ascertain the mechanism of action of the intervention. A larger scale trial is required with a sample size sufficient to group children into clusters based on their profiles and response to the intervention to facilitate ascertainment of the aspects of EPIC that are most strongly associated with improvements in cognition, learning and behaviour. Given the high rates of co-occurrences of children who were recruited on to an ADHD assessment pathway in the current study, we plan to recruit children through community settings in a follow-on full-scale large trial. EPIC principles and practices can be applied generally to neurodivergent children. The current data show the suitability of EPIC for children with co-occurring symptoms.

### Implications for research

These findings add to the emerging evidence base which shows the benefits of psychoeducation and supports a transdiagnostic approach to interventions with neurodivergent children. By employing an individualised approach to identify and support a child’s strengths and weaknesses, we were able to develop an intervention which not only was acceptable and feasible to young people, their teachers and parents but also seemed to be effective in improving aspects of cognition which are particularly of benefit. Further research using a definitive randomised controlled trial will add to this evidence base.

## Data Availability

The data that support the findings of this study are available from first author Sinead Rhodes but ethics restrictions apply to the availability of these data, which were used under license for the current study, and so are not publicly available.

## Footnotes

1 Language statement: There is no unified consensus within the neurodivergent community about language use for condition names [1]. Out of respect to all preferences within this community, including the children, young people and families we work with, ‘with autism’ and ‘autistic’ will be used interchangeably in the current paper.

2 Booklets available here: https://www.ed.ac.uk/clinical-brain-sciences/research/epic-edinburgh-psychoeducation-intervention/epic-resources
